# Acoustic impact of a wave energy converter in Mediterranean shallow waters

**DOI:** 10.1038/s41598-019-45926-1

**Published:** 2019-07-03

**Authors:** Giuseppa Buscaino, Giuliana Mattiazzo, Gianmaria Sannino, Elena Papale, Giovanni Bracco, Rosario Grammauta, Adriana Carillo, Jose Maria Kenny, Norma De Cristofaro, Maria Ceraulo, Salvatore Mazzola

**Affiliations:** 1 National Research Council – Bioacousticslab Capo Granitola, Institute of Anthropic Impact and Sustainability in marine Environment, Via del Mare, 6 – 91021 Torretta Granitola, Campobello di Mazara, (TP) Italy; 2 Department of Mechanical and Aerospace Engineering (DIMEAS), Politecnico di Torino, Corso Duca degli Abruzzi, 24, Torino, TO Italy; 3 ENEA – Climate Modelling and Impacts Laboratory (SSPT-MET-CLIM), via Anguillarese 301, Roma, Italy; 4 Department of Life Sciences and Systems Biology, University of Torino, Via Accademia Albertina 13, 10123 Torino, Italy; 5 University of Perugia, Civil and Environmental Engineering Department, UdR INSTM, Strada di Pentima, 4, Terni, Italy; 6 Universidad Tecnológica Nacional (UTN-FRCH), Puerto Madryn, Argentina

**Keywords:** Boreal ecology, Ecology

## Abstract

In this study, underwater noise from a full-scale wave energy converter system (ISWEC), installed on the coast of Pantelleria Island (central Mediterranean Sea), was characterized. The noise was measured using an autonomous acoustic recorder anchored to the sea bottom 40 m from the ISWEC hull. Acoustic monitoring continued for 15 months, starting 7 months before (PRE), 2 months during (INST) and 6 months after the ISWEC installation (POST). The levels of noise, assessed with power spectrum density and octave and third-octave band sound pressure levels (BSPLs), were higher during the POST period than during the PRE period at lower frequencies up to 4 kHz and increased with wave height. During the ISWEC activation for energy production (POST_ON) in the wave height range 1–2.9 m, the BSPLs increased much more at lower frequencies up to 4 kHz (the median BSPLs at 63 Hz for the PRE, POST, and POST_ON conditions were 73, 106, and 126 dB re 1μPa, respectively). Considering the biophonies that make up the soundscape of the area, we examined the possible masking of fish choruses due to ISWEC noise and highlighted that at a distance of 1000 m, the 800 Hz peak frequency was 10 dB above the ISWEC signal. Within this distance from ISWEC, a possible masking effect is supposed.

## Introduction

In recent decades, the world consumption of energy has significantly increased^1^, and the increase is estimated to continue considerably into the future. In the European Union, the need for pollution-free production processes is forcing the development of renewable energy. In the framework of the dynamic growth of the energy industry, innovative technologies contribute to the exploitation of resources such as biomass, waste, water, wind, geothermal, solar, wave and tidal power. Among these resources, the production of energy from waves is emerging as a clean and constant process: sea waves have the highest energy density among renewable sources^2^.

This increasing demand has led to the construction and installation of a series of infrastructures in the shallow waters off European coasts, especially near islands where energy is more expensive than the continental areas.

The conversion of kinetic energy into electrical energy by wave devices is usually considered to have a low impact on the environment^3^. Although this is true with respect to CO_2_ emissions (no CO_2_ is produced during the wave-energy conversion process), little has been studied regarding the effects of the noise and vibration of the converter equipment, the electromagnetic fields created, the possible disruption of biota and habitats, and water quality changes^3^. A number of wave energy converter prototypes have been developed in recent years, exploiting different energy extraction technologies^4^, deployments and moorings, but only a few specific studies have been carried out on the noise radiated by the different sections of the infrastructure and the potential stress that is inflicted on marine organisms^5^. Even though a number of underwater species use sound to perform various activities (e.g., fish orientate themselves by hearing environmental sounds^6^ and produce sounds during mating periods^7^, and dolphins use sound for numerous key life activities^8,9^), the impact of the noise produced by wave energy converter technologies has been evaluated only for marine mammals^5,10^.

Noise associated with marine traffic, seismic surveys, and sonar and other offshore activities has been found to affect fish, crustaceans, and marine mammals^11–19^. The European Union Marine Environment Strategy Framework Directive (MSFD) (2008/56/EC) is striving for the achievement of an environmental status with good quality for European waters by 2020. In particular, the descriptors of MSFD 11.1 and 11.2 regarding continuous low-frequency sounds and impulsive sounds, respectively, aim to monitor the ambient noise level within the third-octave bands and implement a recording system for temporal-spatial data arising from impulsive anthropogenic sources^20^.

Fish species perceive and use a limited acoustic space for reproduction, communication, and anti-predatory strategies. Acoustic pollution in the frequency less than 2000 Hz can mask signals, compromising intra- and inter-specific communication, individual fitness and causing behavioural and physiological effects that can alter vital functions^11,21–24^. Richardson *et al*.^25^ proposed an impact zone subdivision for anthropogenic sources: zones of audibility, responsiveness, masking, and injury. Noise above the ambient level could cause masking, interfering with a the ability of a listener to detect or recognize another sound^26,27^. In humans, the greatest masking of communication signals occurs when the frequency spectra of the speech signal and the noise are very similar. The effect that results from the masking of a complex sound is particularly challenging to assess in animals^26^. However, in understanding potential auditory masking and in case other information is missing (i.e., an audiogram of the listener, or results of an experimental test based on behavioural and physiological reactions to study the real detection, discrimination, and recognition of the sound), the power spectrum model, with the level of the manmade noise and the signal the animal is trying to hear, is important for determining how detrimental a certain noise is for an animal^26^.

In the sea where the ambient noise levels are critically related to areas in close proximity to shipping lanes, a high number of vessel passages generates intermittent low-frequency noise that, overlaps in frequency with fish signals below 2000 Hz^28^, therefore potentially interfering with fishes detection of ecologically relevant natural acoustic cues.

Within the Mediterranean Sea, which is one of the noisiest basins of the world^29^, the Strait of Sicily is characterized by high levels of both inshore/offshore human activities, biodiversity and endemism^30^. It represents a key habitat for adults, juveniles, and larvae of a number of species and a crossroad for species of Atlantic or Indo-Pacific origins. Therefore, the measurement of noise from new wave energy devices in the shallow waters of this area becomes a crucial issue for evaluating the possible impact on the ecosystem.

In this study, we characterized the contribution of noise in the sea generated by an inertial sea wave energy converter prototype (ISWEC, Wave for Energy srl, Turin, Italy) installed in the waters off Pantelleria Island and analysed the potential acoustic impact on the Mediterranean shallow water ecosystem, considering species that constitute the soundscape.

Because waves are at the base of the infrastructure functioning and the sea state affects the level of noise, we used a forecast system to assess the hourly wave conditions. At very shallow waters near the coast, the sound level increases up to 2000 Hz with wave height^28,31^; therefore, we analysed the noise generated by the ISWEC considering a variation in the wave height and also the status of the ISWEC running at different speeds (rpm) but with the same wave height. We performed a comparison among these 4 conditions: PRE = before the installation of the ISWEC; INST = during the installation of the ISWEC; POST = after the installation and POST_ON = after the installation and during the electrical energy production. Then, we assessed the potential acoustic masking of communication signals of fish species (most probably belonging to *Scopenidae* family^32^ or *Terapon theraps*^33,34^) due to the ISWEC and used a propagation model to determine the distance at which this masking could be considered negligible.

## Results

### Noise levels before and after the ISWEC installation

**Figure 1:**
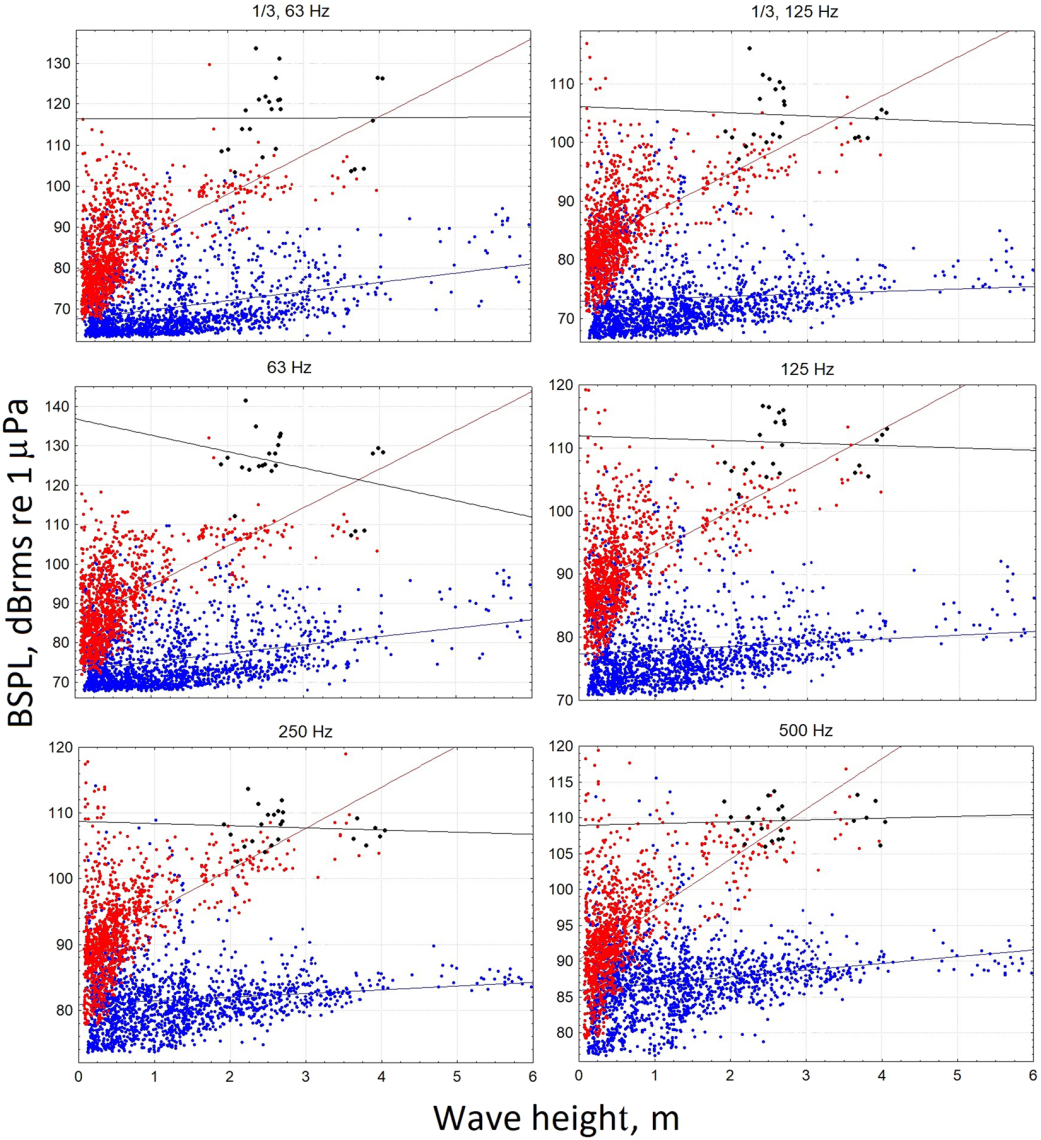
Scatter plots for the band sound pressure level (BSPL, dB_rms_ re 1 μPa) versus wave height at different frequencies in octave bands from 1/3 63 Hz up to 500 Hz. Each point represents the mean BSPL value calculated for each file 2 minutes long. The blue and red points indicate the BSPL values before (PRE, n = 1708) and after (POST, n = 1120) the ISWEC installation, respectively. The black points indicate recordings during the energy conversion (POST_ON, n = 24). The lines represent the linear regression equations.

**Figure 2:**
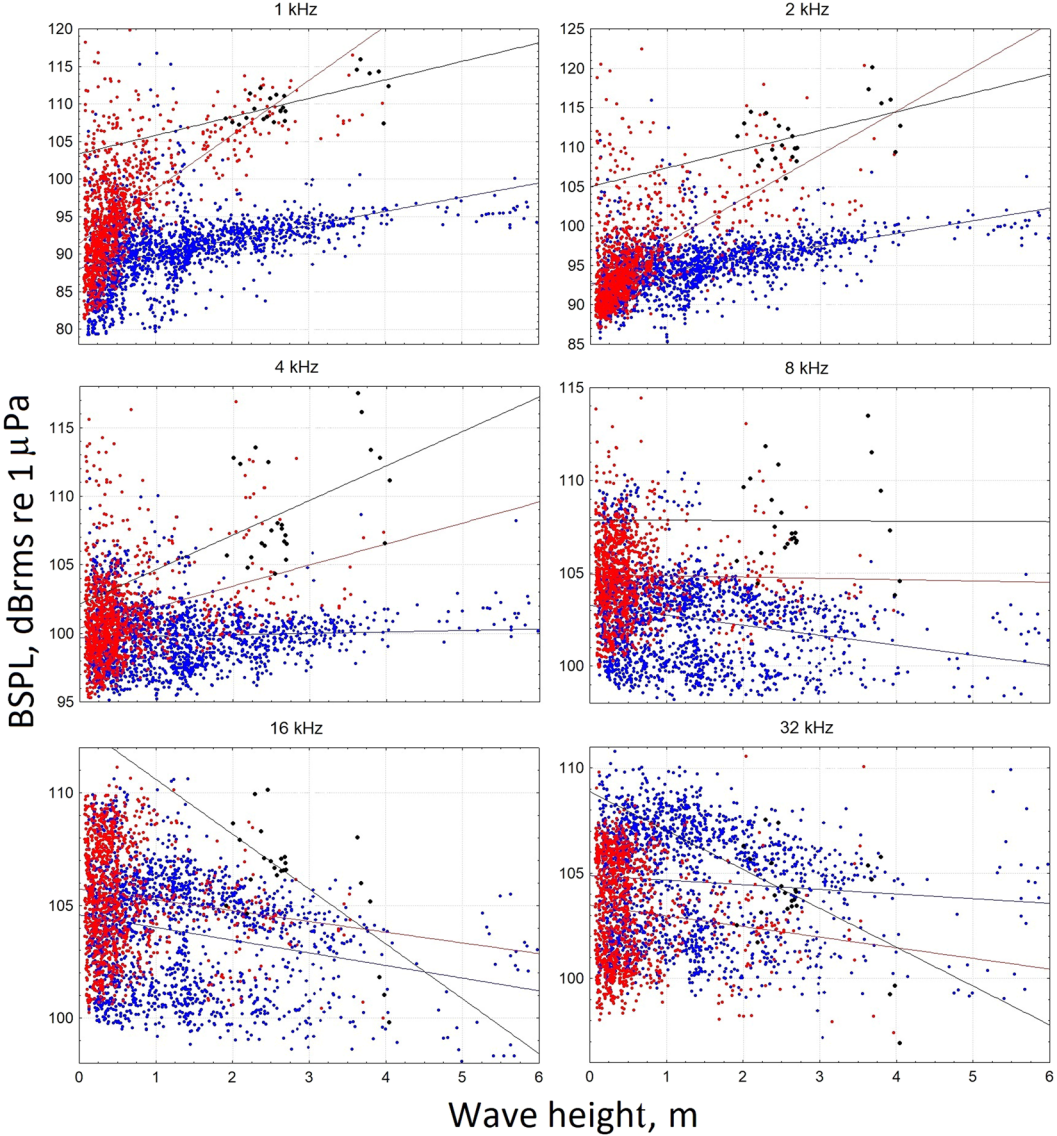
Scatter plots for the octave band sound pressure level (BSPL, dB_rms_ re 1 μPa) versus wave height for frequencies from 1 kHz up to 32 kHz. Each point represents the mean BSPL value calculated for each file 2 minutes long. The blue and red points indicate the BSPL values before (PRE, n = 1708) and after (POST, n = 1120) the ISWEC installation, respectively. The black points indicate recordings during the energy conversion (POST_ON, n = 24). The lines represent the linear regression equations.

Table 1 shows the correlations between BSPLs versus wave height for all data set and for different conditions (PRE, INST, POST, POST_ON). In Figs 1, 2, multiple scatterplots of the BSPLs versus wave height for the PRE, POST and POST_ON conditions are displayed. Excluding the POST_ON condition, BSPLs were positively correlated with wave height up to 4 or 8 kHz (Fig. 1, blue line and Table 1). At higher frequencies, the correlation between wave height and BSPL became weakly negative (except for the INST condition) with generally low values (Fig. 2 and Table 1).

**Table 1:** Spearman Rank Order Correlations values for the octave band sound pressure level (BSPL, dB re 1 µPa) versus wave height (WH) for all data set and for each conditions (PRE: before the ISWEC installation; INST: during the ISWEC installation; POST: after the ISWEC installation; POST_ON: after the ISWEC installation and energy conversion activation). Bold values of correlations are significant at p < 0.05.

BSPL Condition	1/3 63 Hz	1/3 125 Hz	63 Hz	125 Hz	250 Hz	500 Hz	1 kHz	2 kHz	4 kHz	8 kHz	16 kHz	32 kHz	64 kHz
Wave Height - ALL data	0.03	**-0.10**	0.02	**-0.11**	**-0.06**	**0.14**	**0.36**	**0.52**	**-0.04**	**-0.28**	**-0.20**	**0.08**	**0.24**
Wave Height - PRE	**0.31**	**0.16**	**0.27**	**0.16**	**0.15**	**0.24**	**0.53**	**0.57**	**0.05**	**-0.21**	**-0.17**	−0.04	**0.10**
Wave Height - INST	**0.52**	**0.50**	**0.48**	**0.49**	**0.52**	**0.59**	**0.61**	**0.47**	**0.20**	**0.14**	**0.08**	0.01	−0.01
Wave Height - POST	**0.56**	**0.55**	**0.55**	**0.54**	**0.52**	**0.56**	**0.60**	**0.58**	**0.24**	0.00	−0.06	**-0.08**	−0.06
Wave Height - POST_ON	0.19	0.09	0.10	0.10	0.09	0.02	**0.46**	0.24	0.29	−0.08	**-0.44**	−0.28	−0.13

**Figure 3:**
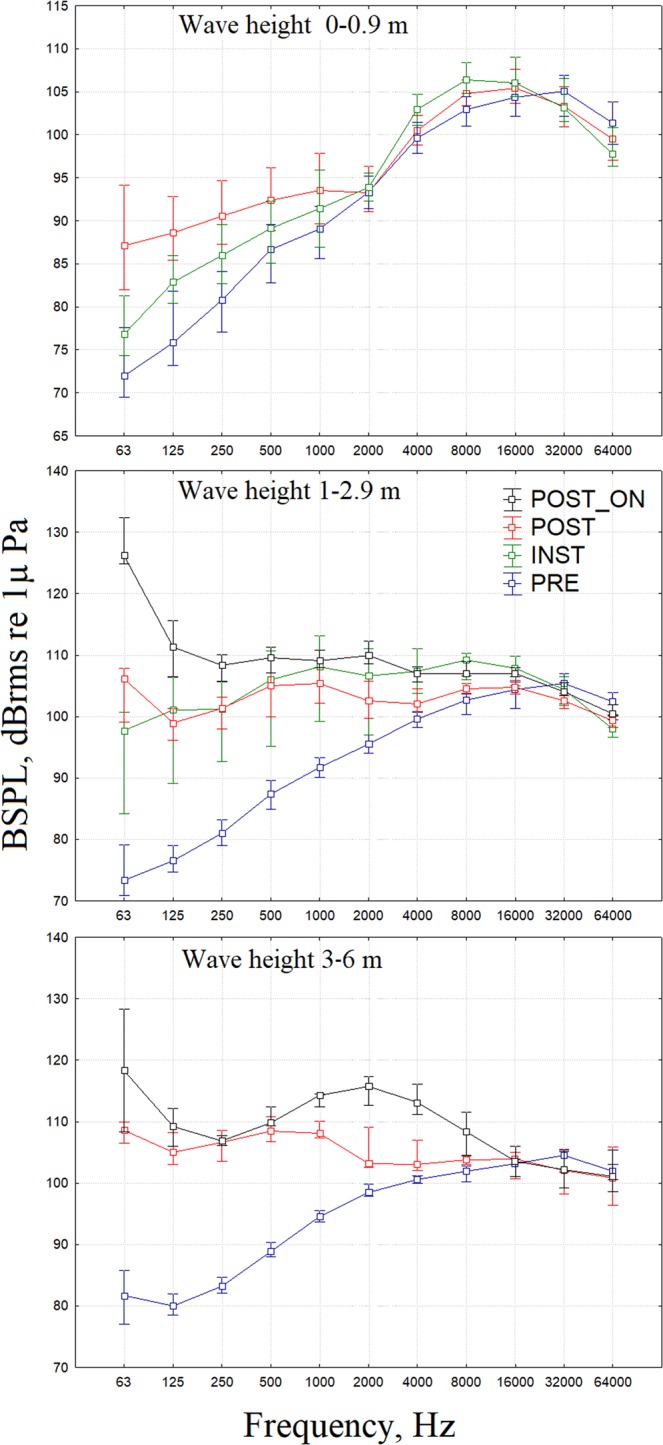
Median (±25^th^ and 75^th^ percentiles) octave band sound pressure level (BSPL, dBrms re 1μPa, 40 m) for different ISWEC conditions (PRE, INST, POST, POST_ON) and for the three wave height ranges (0–0.9, 1–2.9, 3–6 m).

Table 2 shows the median (25^th^–75^th^ percentiles) BSPLs for different condition considering three wave height ranges (0–0.9 m, 1–2.9 m, and 3–6 m) and results of Kruskal-Wallis multiple comparison test to assess significant differences in BPLs between condition (PRE, INST, POST, POST_ON). The differences are all significant for the lower frequencies up to 1000. In Fig. 3, the median (25^th^–75^th^ percentiles) BSPLs are plotted for all conditions (PRE, INST, POST, POST_ON) and for the three wave height ranges. The differences in the BSPLs among the conditions decrease at higher frequencies (Fig. 3). For the 1/3 BSPLs at 63 and 125 Hz (the measurements required to monitor marine noise in the European Marine Strategy Directive - Descriptor 11), the median values (25^th^–75^th^ percentiles) for the intermediate wave height range (1–2.9 m) are 68 (66–73) and 72 (70–74) (condition PRE) and 119 (109–121) and 105 (101–109) (condition POST_ON), respectively (Table 2). Above 4 kHz, the differences in the BSPLs for different conditions decrease, and all the median values are between 100 and 110 dB (Fig. 3).

**Table 2:** Median (25^th^–75^th^ percentiles) BSPLs and Kruskal-Wallis multiple comparison test results for different condition (PRE, INST, POST, POST_ON) within data set divided in three wave height ranges (0–0.9 m, 1–2.9 m, and 3–6 m). ***P-value < 0.0001, **P-value < 0.01, *P-value < 0.05.

Condition (no. cases)		Wave height 0–0.9 m			Wave height 1–2.9 m				Wave height 3–6 m		
		PRE (847)	INST (660)	POST (988)	PRE (740)	INST (108)	POST (122)	POST_ON (18)	PRE (121)	POST(10)	POST_ON (6)
63 Hz	PRE		***	***		***	***	***		***	**
	INST	***		***	***		*	*			
	POST	***	***		***	*			***		
	POST_ON				***	*			**		
	median, 25–75th	72, 70–78	77, 74–81	87, 82–94	73, 71–79	98, 84–101	106, 99–108	126, 125–132	82, 77–86	109, 107–110	118, 108–128
125 Hz	PRE		***	***		***	***	***		***	**
	INST	***		***	***						
	POST	***	***		***				***		
	POST_ON				***				**		
	median, 25–75th	76, 73–82	83, 80–86	89, 85–93	77, 75–79	101, 89–106	99, 96–101	111, 107–116	80, 79–82	105, 103–108	109, 106–112
250 Hz	PRE		***	***		***	***	***		***	**
	INST	***		***	***						
	POST	***	***		***				***		
	POST_ON				***				**		
	median, 25–75th	81, 77–84	86, 83–90	91, 87–95	81, 79–83	101, 93–106	101, 98–103	108, 106–110	83, 82–85	107, 104–109	107, 106–108
500 Hz	PRE		***	***		***	***	***		***	**
	INST	***		***	***						
	POST	***	***		***				***		
	POST_ON				***				**		
	median, 25–75th	87, 83–90	89, 85–92	92, 89–96	87, 85–90	106, 95–111	105, 100–107	110, 106–110	89, 88–90	108, 107–111	110, 109–112
1000 Hz	PRE		***	***		***	***	***		***	**
	INST	***		***	***						
	POST	***	***		***				***		
	POST_ON				***				**		
	median, 25–75th	89, 86–92	91, 87–96	94, 90–98	92, 90–93	108,99–113	105, 102–108	109, 108–111	95, 94–96	108, 107–110	114, 112–115
2000 Hz	PRE		***			***	***	***		***	***
	INST	***		**	***						
	POST		**		***				***		
	POST_ON				***				***		
	median, 25–75th	93, 91–95	94, 92–96	93, 91–96	96, 94–97	107, 97–111	103, 100–106	110, 109–112	98, 98–100	103, 103–109	116, 113–117
4000 Hz	PRE		***	***		***	***	***		***	**
	INST	***		***	***		**				
	POST	***	***		***	**		*	***		
	POST_ON				***		*		**		
	median, 25–75th	99, 98–101	103, 101–105	100, 99–102	100, 100–104	107, 104–111	102, 101–105	107, 106–108	101, 100–101	103, 102–107	113, 111–116
8000 Hz	PRE		***	***		***	***	***		**	**
	INST	***		***	***		***				
	POST	***	***		***	***		*	**		
	POST_ON				***		*		**		
	median, 25–75th	103, 101–104	106, 105–108	105, 103–106	103, 100–104	109, 106–110	104, 104–105	107, 107–109	102, 100–103	104, 103–104	108, 105–112
16000 Hz	PRE		***	***		***	**	***			
	INST	***		***	***		***				
	POST	***	***		**	***		**			
	POST_ON				***		**				
	median, 25–75th	104, 102–106	106, 104–109	105, 104–108	104, 101–106	108, 105–110	105, 104–106	107, 107–108	103, 101–104	104, 101–105	104, 101–106
32000 Hz	PRE		***	***			***				
	INST	***		***			***				
	POST	***	***		***	***					
	POST_ON										
	median, 25–75th	105, 102–107	103, 102–107	103, 101–106	105, 102–107	104, 102–107	103, 101–104	104, 103–105	105, 102–105	102, 98–105	102, 99–105
64000 Hz	PRE		***	***		***	***				
	INST	***		***	***			*			
	POST	***	***		***						
	POST_ON					*					
	median, 25–75th	101, 99–104	98, 96–101	100, 97–101	102, 99–104	98, 97–100	99, 98–100	100, 100–102	102, 101–103	101, 96–106	101, 99–105
1/3 63 Hz	PRE		***	***		***	***	***		***	***
	INST	***		***	***						
	POST	***	***		***				***		
	POST_ON				***				***		
	median, 25–75th	67, 65–71	71, 69–76	81, 76–88	68, 66–73	94, 79–98	98, 93–100	119, 109–121	76, 72–81	102, 99–102	110, 104–126
1/3 125 Hz	PRE		***	***		***	***	***		***	***
	INST	***		***	***						
	POST	***	***		***				***		
	POST_ON				***				***		
	median, 25–75th	71, 69–77	78, 75–81	83, 80–87	72, 70–74	98, 85–104	94, 91–96	105, 101–109	75, 74–77	100, 98–103	103, 101–105

**Figure 4:**
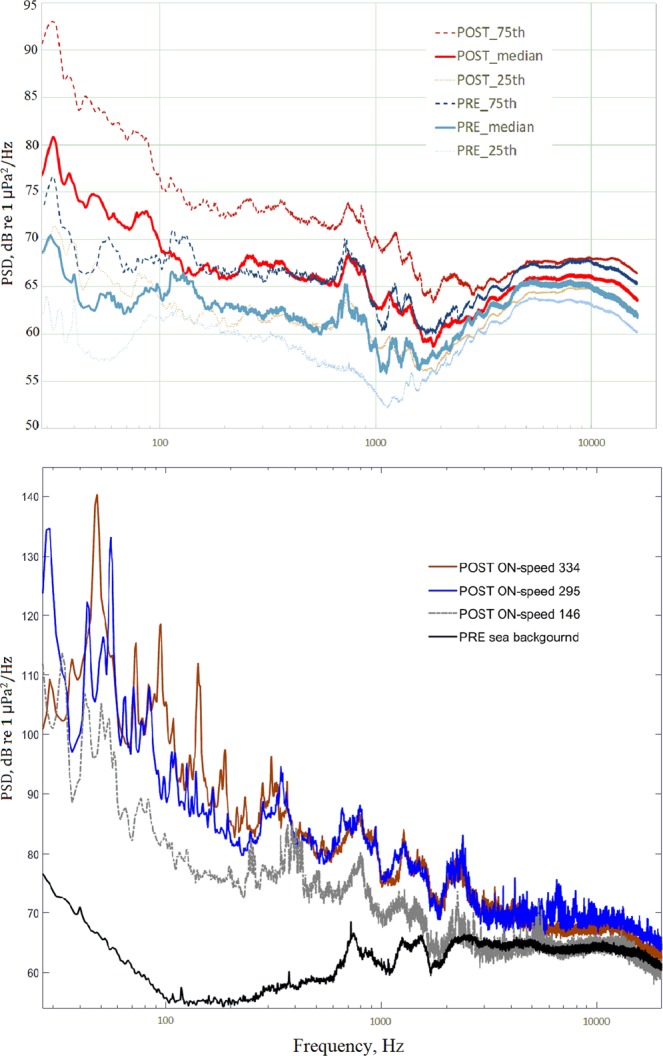
TOP: Median (25^th^–75^th^ percentile) power spectral density (PSD, dB re 1 μPa^2^/Hz, 40 m distance) for PRE and POST condition (period 30 May–30 November 2015). BELOW: Power spectral density (PSD, dB re 1 μPa^2^/Hz, 40 m distance) of recordings with the ISWEC infrastructure during energy production (ISWEC_Post_ON, with a wave height range of 2.2–2.4 m, 20 November 2015, time 16:00–23:00; PRE - Sea background noise, 12 December 2014, time 16:00, wave height 2.3 m) at different flywheel speeds.

Figure 4 shows the power spectral density (dB re 1 μPa^2^/Hz, 40 m distance) of PRE and POST condition (Fig. 4, Top) and the ISWEC during energy conversion at different flywheel speeds (Fig. 4, Below). The peak frequency and its amplitude change with the speed of the flywheel. The peak frequencies are below 50 Hz and reach 140 dB for a flywheel speed of 334 rpm. The sound pressure levels at different flywheel speeds of 334, 236 and 146 rpm are 143, 137, and 128 dB (SPL, dB_rms_ re 1 μPa, 40 m distance, a wave height of 2.3 m, and a frequency band of 20–96000 Hz). The source levels (SL, dB_rms_ re 1 μPa, 1 m distance, a wave height of 2.3 m, and a frequency band of 20–96000 Hz) corresponding to these flywheel speeds were assessed using the transmission loss model and are 173, 167, and 158 dB, respectively.

### ISWEC masking of fish choruses

**Figure 5:**
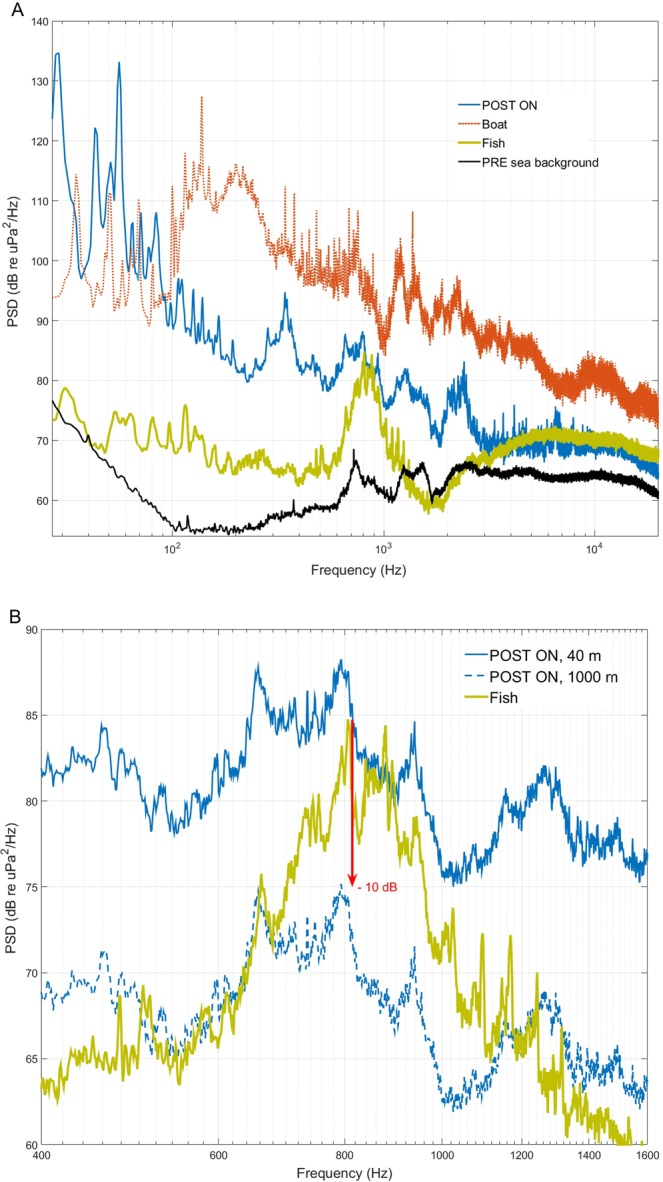
(**A**) Power spectral density (dB re 1 μPa^2^/Hz) of recordings with the ISWEC infrastructure during energy production (ISWEC_Post_ON, with a wave height of 2.2 m, 20 November 2015, time 17:30, recorded at a distance of 40 m, flywheel speed 295); a boat passage (Boat) (30 June 2015, time 09:30); acoustic fish activity most probably belonging to *Scopenidae* family^32^ or *Terapon theraps*^33,34^ (28 June 2015, time 21:05); sea background noise (wave height of 2.1 m, 25 January 2015, time 15:00). (**B**) Zoom of the above graph, which also shows the PSD of ISWEC POST_ON at a 1000 m distance, which corresponds to a decrement of approximately 13 dB at 800 Hz and is 10 dB below the fish choruses peak.

Figure 5A shows the PSD calculated for different conditions (ISWEC_Post_ON speed of 295 rpm, fish choruses, background noise and an example of a boat passage). The energy of the fish choruses is associated with frequencies between 600 and 1100 Hz. During energy conversion, at the peak frequency of the chorus (800 Hz), the ISWEC PSD is higher than the PSD of the fish at approximately 4 dB (see the zoom in Fig. 5B). However, the noise produced by a boat passage is higher than that of both the ISWEC and fish choruses from 100 Hz up to 10 kHz (Fig. 5A).

Implementing the transmission loss model for the ISWEC Post_ON condition, we found that at a distance of 1000 m from the ISWEC (Fig. 5B), the noise generated is strongly reduced (see blue dashed line). In detail, at that distance, the PSD of the noise produced by the ISWEC during energy conversion has a decrement of 13 dB at 800 Hz and is 10 dB below the peak frequency of the fish choruses (Fig. 5B).

## Discussion

Wave energy devices and the associated cables could act as a potential source of cumulative stressors on aquatic ecosystems^23,35^. Anthropogenic activities can increase ambient noise^23,26^, and operating wave energy converters contribute to vibrations and low-frequency long-duration noise. Because ambient noise increases with increasing wind speed and wave height and wave energy converters act with moving water masses, we first evaluated the increase in noise in relation to wave height. We found that noise increases more with wave height during the mooring installation (INST) and in the presence of the ISWEC infrastructure (POST) than without the ISWEC (PRE) (see Table 1 and Fig. 3). This is probably due to the anchoring system consisting of metallic chains that are more stimulated by higher waves (Figs 7, 10). In the presence of the ISWEC infrastructure (POST), the level of noise is higher than the background noise (PRE), for frequencies up to 4 kHz. Above 4 kHz, the differences between BPLS (PRE vs POST) decrease even if they are still significant (see Fig. 3 and Table 2).

The process of energy conversion (Post_ON) provided additional noise compared to the POST condition at lower frequencies up to 8 kHz, with a maximum difference of 20 dB for the 63 Hz BSPL (see Fig. 3 and Table 2). Therefore, the noise of the infrastructure is produced both by the vibrating bulkhead with its anchoring system and by the activation of the energy conversion system. During energy conversion (POST_ON), the noise changes with flywheel speed, with a maximum SPL corresponding to the maximum speed considered (334 rpm; SPL 143 dB_rms_ re 40 m and a peak frequency of 140 dB at 50 Hz; see Fig. 4).

On Pantelleria Island (Fig. 8), the ISWEC was installed through a complex chain system to maintain a steady infrastructure. The noise generated by this system occupies frequency bands up to 8 kHz (see Figs 3, 10). Instead, noise emitted during energy conversion, likely generated by the hydraulic pump and gyroscopic units, overcomes the ambient noise by at least 53 dB at the frequency of the 63 Hz BSPL (see in Fig. 3 and Table 2 the differences between the PRE and POST ON conditions, wave height range 1–2.9 m).

A number of recent works have shown that noise recorded from wind turbines and wave energy converters is audible by marine species such as fish^36,37^, crustaceans^38,39^, and pinnipeds^5^, but this noise is probably out of the audible zone of toothed whales^5,40^. The effects of the generated noise depend on the sensitivity of each species, their ability to habituate to the noise, and their behavioural state. In this work, the position of the ISWEC near the coastline and harbour is probably outside the migration routes of whales^41^, and the noise generated by the ISWEC overlaps with high-intensity fish choruses occurring in the summer season. Whalberg *et al*.^37^ highlighted that the extent of the impact of sound pressure or the relative particle motion on fish communication, behaviour and fitness is still unknown. Considering the potential communicative function of these sounds^32^, the masking of fish signals might have consequences at the individual and population levels (i.e. the period during which choruses were recorded could correspond to courtship and spawning).

In our recordings, the fish chorus peak frequency (800 Hz) was overcome by more than 3 dB by the ISWEC noise (Fig. 5). Then, by using the transmission loss model, we highlighted that at a distance of 1000 m, the ISWEC noise was reduced 10 dB below the fish chorus peak at 800 Hz (Fig. 10). However, the PSD measured for a vessel passage was much more intense at all frequencies (except frequencies lower than 100 Hz), overcoming a fish chorus of approximately 20 dB. Even though in this study we could not evaluate whether the ISWEC noise affects the perception of conspecific sounds in fish, our results suggest that the noise of the ISWEC during its activity (POST_ON condition) can have an effect within a 1000 m radius. This is a first step study based on a simple power spectrum model, and further information will be necessary to measure the masking effect regarding fish calls^26,42^. Indeed, in order to determine masking effects of anthropogenic noise, more baseline studies should focus on hearing abilities (i.e. audiograms, SNR required for detection, discrimination, recognition of conspecific calls) and the communication space (effective vocalization radius) of fish species^26^.

In conclusion, underwater noise radiated from a full-scale wave energy converter system (ISWEC) was assessed in the shallow waters near the coast of Pantelleria Island in the central Mediterranean Sea. The noise of the ISWEC is higher at lower frequencies up to 4 kHz, especially when the ISWEC is active for energy production. The noise arises from both the anchoring system and the hull during energy conversion. The noise of the ISWEC infrastructure increases with wave height. ISWEC noise power spectrum amplitude exceeds that of the fish chorus power spectrum, therefore it is potentially masking fish choruses and, according to a transmission loss model, at a distance of 1000 m the fish peak is 10 dB higher than the ISWEC noise. Improvements in the materials in the anchoring system and in the bearings of moving parts could reduce the ISWEC noise and the noise of wave energy converter systems in general. Moreover, in steady operation for energy production, scheduled interruptions could be planned to avoid masking of fish choruses during dusk in the summer season, which could corresponds to courtship and spawning time.

## Materials and Methods

### ISWEC description

**Figure 6:**
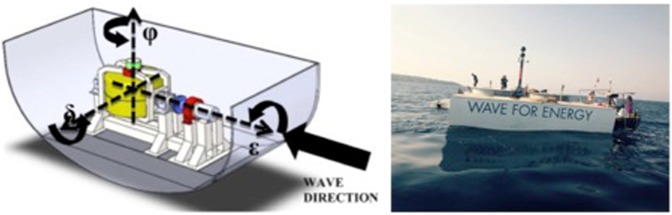
Scheme of the ISWEC (left) and a photo of the ISWEC deployed at Pantelleria (right).

The ISWEC (inertial sea wave energy converter) is a floating all-enclosed converter retained by a slack mooring (Fig. 6 and Table 3). The electro-mechanical system is completely sealed into the central body, granting intrinsic reliability and reduced maintenance. Wave power extraction is obtained by inertial torques provided by a gyroscope on an internal precession axis. An electric PTO (power take off) is connected to the precession motion, converting mechanical energy into electrical energy.

**Figure 7:**
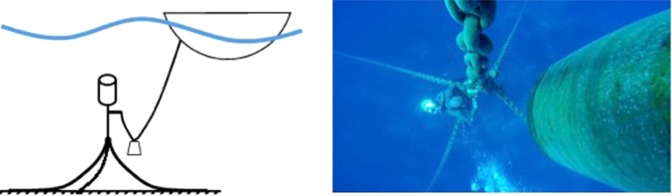
Scheme of the mooring (left) and a photo of the system from the sea surface (four chain junction and mass).

**Figure 8:**
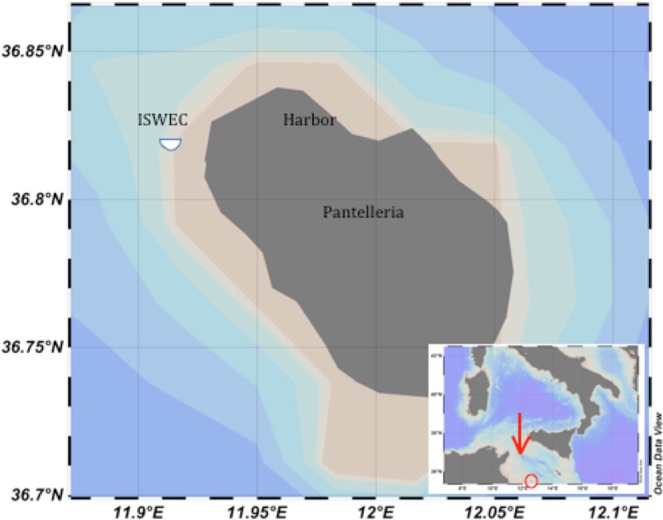
Study area. The red arrow and circle indicate Pantelleria and Lampedusa Islands, respectively. (Map source: Schlitzer, R., Ocean Data View, odv.awi.de, 2015).

**Figure 9:**
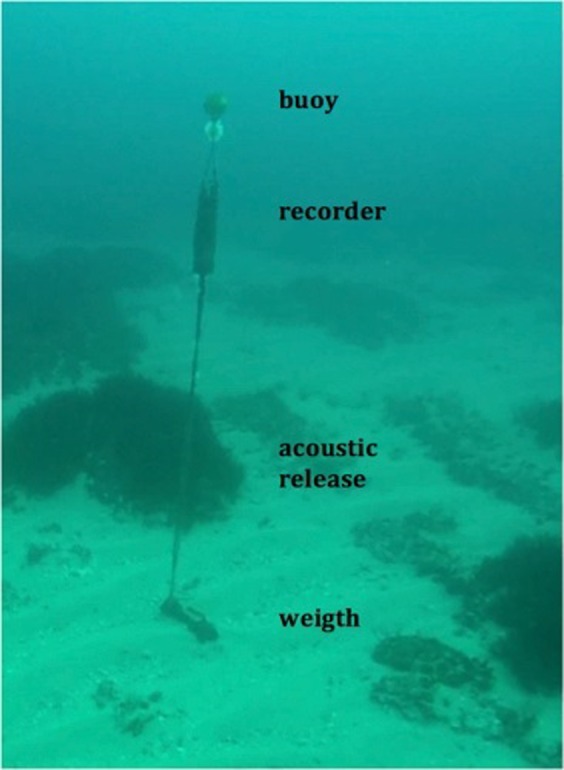
Mooring of the underwater acoustic recorder positioned at the sea bottom with a mix of Mediterranean seagrass, sand and rocks.

**Figure 10:**
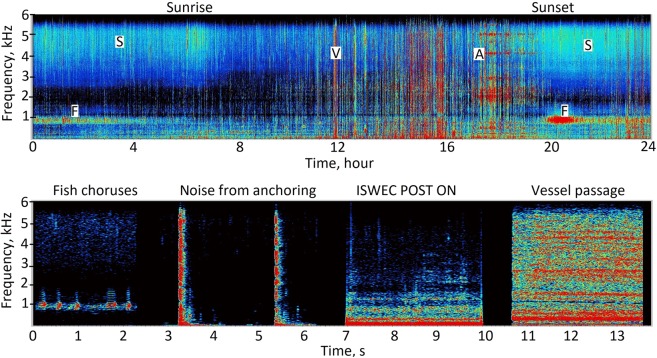
Top: One-day continuous spectrogram (6 September 2015) showing the snapping shrimp sound (S), fish choruses (F), anthropogenic noise caused by the passage of vessels (V), and the noise due to the (A) anchoring system of the ISWEC. The mean (±standard deviation) wave height was 0.70 ± 0.06 m. Below: Fish choruses, noise from the anchoring system (POST), noise from the ISWEC activated for energy conversion (Post ON) and noise from a vessel passage (these sounds were composed in sequence in a unique file and are separated by 1–2 s of silence). x-axis: time; y-axis: frequency in kHz, and the SPL intensity is shown in colour scale. For both figures, spectrograms were obtained using SASLab software (Avisoft, Germany) (FFT length of 1024, with a Hamming window, time segments overlap 75%). x-axis: time; y-axis: frequency on a linear scale.

**Table 3:** ISWEC main parameters.

Parameter	Value	
Total mass	316	ton
Ballast mass	200	ton
Floater length	15	m
Floater width	8.05	m
Floater height	5	m
Flywheel maximum speed	400	rpm
Flywheel moment of inertia	7.5∙10^3^	kgm^2^
Flywheel mass	10	ton

The ISWEC, deployed at Pantelleria in August 2015, is a device with a rated power of 100 kW and consists of a steel hull carrying two independent gyroscopic units (Fig. 6). Each unit consists of a flywheel inside a vacuum chamber to reduce the spinning losses. The vacuum chamber is designed to sustain the gyro and transfer its actions to the PTO. The gyro is supported by two radial roller bearings for the radial load and spherical roller thrust bearing for the axial load. Due to the relevant loading conditions, the bearings are provided with an oil cooling and lubrication system.

The flywheel rotation requires energy, and its speed is controlled by software to increase the ratio between energy production (depending on the wave conditions) and energy absorption. However, considering the aims to evaluate the isolated contribution of different flywheel speeds to the generated noise, we used different flywheel speeds with the same wave height.

### ISWEC mooring system

The ISWEC is equipped with three mooring lines (Fig. 7): a main mooring line on the fore for the wave actions and two mooring lines on the sides to keep the device aligned with the main wave direction and restrict the yaw to avoid the electrical cable connection. In the installation considered in this work, the only mooring line present was the main mooring line, since during the first deployment, there was not an electrical connection to the grid. It is plausible expects an increase of noise coming from additional metallic chains if all three mooring lines are present.

The main mooring line provides the necessary compliance for normal operation and simultaneously restrains the device position in the area. The mooring line is constituted by four anchors (hall-type) positioned on the seabed in a circle of diameter of 100 m. A chain departs from each anchor, and the free ends of the four chains are joined together and pulled upwards by a jumper, which pre-stresses the system, reducing the dragging and abrasion of the seabed. From the jumper, a chain reaches a clump weight, and then the last chain line, ending in a bifurcation, links the weight to the ISWEC.

The main mooring line is a non-linear restraint system that adapts its restraint force to the ISWEC functionality. The mooring exerts small force for moderate to medium wave conditions when the ISWEC energy production is active, and the pitching motion must not be reduced by the mooring forces. On the other hand, the mooring increases its restraint force in extreme weather conditions, where the ISWEC is shut down to prevent damage.

### Study area

The ISWEC was installed approximately 750 m from the North Western Coast of Pantelleria Island (position 36°49′22.2″N – 11°55.0′14.4″E), 55 nautical miles from Cape Granitola (Southern Coast of Italy) and 39 miles from Cape Bon (Northern Tunisia) (Fig. 8). In this area, the sea bottom is not uniform and consists of a mix of Mediterranean seagrass (*Posidonia oceanica*), sand and rocks (Fig. 9).

The Island of Pantelleria is the tip of the deep volcanic Pantelleria Graben^43^ located in the central western side of the Strait of Sicily, a high biodiversity hot spot in Mediterranean Sea^44^, The island is strongly exposed to the prevailing north winds^45^. The deep North-Western Coast satisfies the requirements for a good installation site of a wave energy converter since, in the area, the power of the waves was estimated to be 62.5 MWh/m/y^46^. The island is totally dependent on energy generated through diesel, and the energy costs for the users are double those for the continental areas.

### Acoustic data acquisition

An underwater acoustic recorder was placed at a 25 m depth, with a hydrophone height of 5.0 m from the sea bottom and 40 m from the ISWEC (Fig. 9).

The acoustic data were collected from 24 November 2014 to 25 January 2016. Since the infrastructure was deployed in August 2015, we obtained a set of data before the installation (from November 2014 to June 2015), during the installation of the mooring system and ISWEC (June-August 2015), and with the ISWEC present (from August 2015 to January 2016). We used an autonomous recorder (SM2, Wildlife Acoustics, US) with a ultrasonic hydrophone with a recording bandwidth of 8 to 150000 Hz and a declared sensitivities of −170 ± 5 dB re 1 V/μPa in the band of 25–200 Hz, −166 ± 1 dB re 1 V/μPa in the band of 100 Hz–15000 Hz, and −170 ± 5 dB re 1 V/μPa in the band 15–100 kHz. A 35 kg weight and a small sub-surface buoy were used to maintain the vertical arrangement in the case of strong currents or bad weather (a photo with all components of the mooring is shown in Fig. 9). The buoy was connected to the upper part of the recorder with a thin rope (the distance between the buoy and hydrophone was 2 m). All the components were connected with non-metallic ropes to avoid noise due to moving parts.

We used two sampling strategy. In the fist, used for PSD analysis, we set the sampling frequency to 192000 Hz, we sampled two day over three. In the days of recording we sampled using a duty cycle of 2 minutes for recording (.wav files) and 58 minutes of no recording. For the second sampling strategy, used for the preliminary soundscape analysis, we set the sampling frequency at 48000 Hz and recorded in a continuous way. We sampled one day over three. For both sampling strategies we used a resolution of 16 bits and no pre-amplification or filtering was applied during the recordings (except for the automatic anti-aliasing filter of the recorder).

Using an acoustic releaser, the recorder was recovered for maintenance every 3 months to change the batteries and storage memory. For each maintenance event, recorder was non-operative for two/three days (except for the months of April and October 2015 when the recorder did not work).

### Acoustic analysis

#### Band Sound Pressure Level (BSPL)

For each 2-minute file, the power spectral density (PSD) (dB re 1 µPa^2^/Hz), with Welch’s overlapped segment averaging estimator method^47^, was calculated. The calculation was performed by dividing the acoustic data into Hann windows of 192000 samples (∆t = 1 s), with a signal superposition of 50%. From these PSDs, the sound pressure level (SPL, dB re 1 μPa) and the octave band sound pressure level (BSPL, dB re 1 μPa) were calculated integrating over the specific band frequency. In total, 11 octave frequency (Hz) bands were considered: 63 (44–88), 125 (88–177), 250 (177–355), 500 (355–710), 1000 (710–1420), 2000 (1420–2840), 4000 (2840–5680), 8000 (5680–11360), 16000 (11360–22720), 32000 (22720–45440), and 64000 (45440–90880). This non-linear frequency band partitioning was used to obtain a higher resolution at low frequencies, which showed greater variability than the higher frequencies. Moreover, as mandatory in Descriptor 11 by the European Marine Strategy Framework Directive (2008/56/EC11) for monitoring marine noise, the one-third octave band sound pressure level (BSPL, dB re 1 μPa) at the 63 Hz (56.2–70.8) and 125 Hz (112–141) central frequencies have been calculated^20^.

#### Soundscape Components of the study area

A preliminary visual qualitative analysis of the acoustic recordings (Fig. 10) was conducted on a subsample of 3 days per month (randomly selected) to confirm the presence of the main components of the soundscape already described by Buscaino *et al*.^28^ and Ceraulo *et al*.^48^, for Lampedusa Island (approximately 140 km from Pantelleria island) and for the Sicilian Southwestern Coast, Cape Granitola (approximately 95 km from Pantelleria island), respectively. All these sites in the Sicilian Channel (Fig. 8) present some common characteristics: they are in very shallow waters, near the coast, and have a sea bottom consisting of a mix of Mediterranean seagrass, sand, and rock.

**Figure 11:**
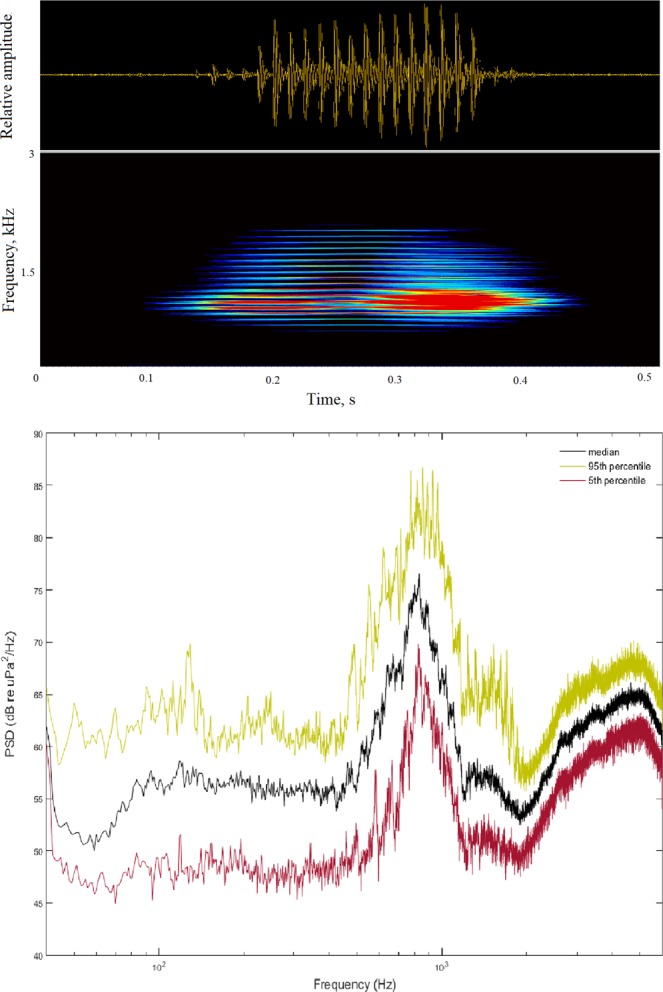
Top: oscillogram and spectrogram showing a pulses train composing a fish call (sampling frequency 6000 Hz, FFT length of 1024, Hamming window, time segment overlap 25%). Below: Median (±95th and 5th percentiles) Power Spectrum Density (dB re 1 μPa^2^/Hz) of fish calls (no. 286) recorded in the period 30 May−18 September 2015 and selected considering the best ratio signal to noise and an homogeneous number of calls for each month.

We found that the measurements at frequencies below 1000 Hz are dominated by noise generated by waves and are louder during the winter; conversely, snapping shrimps dominate the measurements at higher frequencies from 4000 up to 96000 Hz and increase their acoustic activity during the summer. During summer, fish choruses dominate the measurements in the frequency range up to 2000 Hz, especially during dusk and night hours until dawn (Fig. 10). These intense choruses are created by fish signals that mainly occupy a frequency range between 600 and 1200 Hz with a peak frequency of 800 Hz (Figs 5, 10, 11). Their acoustic features and temporal patterns are comparable to the fish signals already described in a Mediterranean Posidonia meadow^32,48^. These signals are also acoustically comparable to fish sounds recorded in shallow water of southwester Arabian Sea^34^ and in the Great Barrier Reef (Australia)^33^. Relying on these precedent studies, fish sounds could be assigned to *Terapon theraps* (for which our study area should be a suitable habitat; see fishbase suitable habitat map) or to a species belonging to *Scorpaenidae* family. A description of the main sound characteristics for our study site is given in Table 4 and in Fig. 11. Anthropogenic noise is represented by vessel passages (Fig. 10).

**Table 4:** Description of acoustic carachteristics of fish calls (no. 286) recorded in the period 30 May–18 September 2015.

	No. of pulses per call	Call duration, ms	Pulse repetition rate, Hz	Pulse peak frequency, Hz
Median	14	194	66	790
25th-7th5 percentile	11–18	126–286	54–72	750–890

#### ISWEC masking of fish choruses

After identifying the soundscape components, we analysed the potential masking of fish choruses by ISWEC noise. We focused our analysis on fish choruses because the peak energy of the ISWEC noise overlaps in frequency with the fish choruses.

Here, we could not evaluate whether the ISWEC noise affects the perception of conspecific sounds by fish (no data are available on the hearing capability of these fish). Therefore, we considered masking as noise overlap when its energy is in the frequency region of the fish signal^26^.

For each file, the power spectral density (PSD)(dB re 1 µPa^2^/Hz) was calculated following Welch’s overlapped segment averaging estimator method^47^. The calculation was performed by dividing the acoustic data into Hamming windows of 192000 samples (∆t = 1 s), with a signal superposition of 50%.

We calculated the PSD on selected files containing (a) the highest intensity of fish chorus signals assumed to be the closest to the hydrophone; (b) ISWEC noise during activation for energy production (ISWEC_Post_ON) with a flywheel speed of 295 rpm (the most suitable speed for the median wave height wave in Pantelleria); (c) sea background noise; and (d) an example of a boat passage.

We hypothesized as acceptable the reduction of the ISWEC noise when the peak frequency of the fish chorus is above the ISWEC noise by at least 10 dB. Based on this result, we applied a transmission loss (TL) model to the total PSD levels recorded for the Post_ON condition to calculate the distance at which the ISWEC noise has a negligible masking effect on the fish signals.

The transmission loss (TL) model was applied to the total PSD levels recorded for the Post_ON condition. We explored the variation in the PSD during noise propagation through the seawater at 40 m (the distance between the ISWEC and the recorder) and at the distance to obtain a negligible masking effect on the fish choruses at 800 Hz. The model considered the attenuation due to geometric spreading and absorption processes related to the dissolved salts^49,50^. The geometric spreading model was used to take into account spherical spreading until the maximum water depth (D = 25 m) and cylindrical spreading for the remaining distance. For ranges R > D, the transmission loss TL becomes:

$$TL=20{\rm{logD}}+10\,\mathrm{log}\,R/D+\alpha {\rm{R}}$$

*where R* is the distance from the source, and α is the absorption coefficient calculated as:

$${\rm{\alpha }}={C}_{1}\frac{{f}_{1}{f}^{2}}{{{f}^{2}}_{1}+{f}^{2}}+{C}_{2}\frac{{f}_{2}{f}^{2}}{{{f}^{2}}_{2}+{f}^{2}}+{C}_{3}{f}^{2}$$

The terms of the formula display the contributions of temperature, hydrostatic pressure and salinity through the relaxation frequencies (f_1_ = 1.848; f_2_ = 151.415) and the coefficient (*C*_1_* = 0*.2*165, C*_*2*_* = 0.8250, C*_3_* = 0.0004)*. We used the mean values of the conductivity, temperature and depth data (CTD) acquired with specific profiles during May and November 2015 at seven sampling points within a radius of 200 m from ISWEC (May measurements: PH = 8.4; S = 37.2‰; T = 18.0 °C; November measurements: PH = 8.4; S = 37.6‰; T = 21.8). The profiles of the absorption coefficient α (dB/km), calculated using the salinity, temperature and PH data obtained in the two measurement periods do not show significant differences.

The evaluation of the PSD at a distance R from the source (ISWEC) was carried out with the following equation:

$${\rm{P}}{\rm{S}}{\rm{D}}({\rm{R}})={\rm{S}}{\rm{L}}{\textstyle \text{-}}{\rm{T}}{\rm{L}}({\rm{R}})$$

where SL is the source level of the ISWEC (dB re 1 µPa^2^/Hz at 1 m), valued with the same geometric spreading model presented above.

#### High-resolution sea state forecast

We used a forecast system to assess the hourly wave conditions and analyse the relationships between the noise generated by the instrument and an increase of wave height conditions (see the statistical analysis paragraph). An operational wave forecast system for the entire Mediterranean basin has been running at ENEA since June 2013. Simulations are performed with a parallel version of the WAM wave model Cycle 4.5.3^51^ at a resolution of 1/32° in each direction, corresponding to a linear mesh size of approximately 3.5 km. A series of nested higher resolution simulations (1/124°) are then performed using SWAN (Simulating WAves Near shore) model^52^. The entire forecast system consisting of WAM and SWAN models is forced with hourly wind fields obtained from the meteorological operational system SKIRON, developed by the Atmospheric Modeling and Weather Forecasting Group of the University of Athens^53^. The atmospheric model is run daily over the Mediterranean basin at a horizontal resolution of 0.05° × 0.05°. The system produces daily wave forecasts for the next 5 days, starting at time 00Z. The directional wave energy density spectrum, both for WAM and SWAN, is discretized using 36 directional bins, corresponding to an angular resolution of 10°, and 32 frequency bins starting from 0.06 Hz with relative size increments of 0.1 between the frequency bins. Significant wave heights produced by the operational system have been validated against data derived from satellite measurements over the period between June 2013 and November 2014^54^. In this paper, we used data derived from the SWAN simulation performed on an area extending for 0.5° in longitude and in latitude around the island of Pantelleria. The grid extends from the point of coordinates 11.75°E, 36.5125°N to the point 12.15°E, 37.0125°N. Hourly significant wave heights from the first day of wave forecasting have been used. (https://giotto.casaccia.enea.it/waves/).

### Statistical analysis

To evaluate the contribution of the ISWEC to the ambient noise level, we first explored data using scatterplot of BPLs vs. wave height at different conditions (PRE, POST, POST_ON). We applied Spearman correlation analysis to verify correlation between wave height and BPLS at different conditions (PRE, INST, POST, POST_ON). Since wave height differentially affected the BSPL values under different conditions, data were not normally distributed and BPLs variance was not homogeneous along the wave height, we performed analysis dividing our data set in ranges of three wave height: 0–0.9 m, 1–2.9 m, and 3–6 m. Within each wave range we used non-parametric Kruskal-Wallis multiple comparison test to assess differences in the BSPLs values between conditions: before (condition: PRE), during the ISWEC infrastructure installation (condition: INST), after the installation of the ISWEC infrastructure (condition: POST), and during the energy conversion of the ISWEC infrastructure (condition: POST_ON).
